# Ten Simple Rules for Reviewers

**DOI:** 10.1371/journal.pcbi.0020110

**Published:** 2006-09-29

**Authors:** Philip E Bourne, Alon Korngreen

Last summer, the Student Council of the International Society for Computational Biology prompted an Editorial, “Ten Simple Rules for Getting Published” [1]. The interest in that piece (it has been downloaded 14,880 times thus far) prompted “Ten Simple Rules for Writing a Grant” [2]. With this third contribution, the “Ten Rules” series would seem to be established, and more rules for different audiences are in the making. *Ten Simple Rules for Reviewers* is based upon our years of experience as reviewers and as managers of the review process. Suggestions also came from PLoS staff and Editors and our research groups, the latter being new and fresh to the process of reviewing.

The rules for getting articles published included advice on becoming a reviewer early in your career. If you followed that advice, by working through your mentors who will ask you to review, you will then hopefully find these *Ten Rules for Reviewers* helpful. There is no magic formula for what constitutes a good or a bad paper—the majority of papers fall in between—so what do you look for as a reviewer? We would suggest, above all else, you are looking for what the journal you are reviewing for prides itself on. Scientific novelty—there is just too much “me-too” in scientific papers—is often the prerequisite, but not always. There is certainly a place for papers that, for example, support existing hypotheses, or provide a new or modified interpretation of an existing finding. After journal scope, it comes down to a well-presented argument and everything else described in “Ten Simple Rules for Getting Published” [1]. Once you know what to look for in a paper, the following simple reviewer guidelines we hope will be useful. Certainly (as with all *PLoS Computational Biology* material) we invite readers to use the PLoS eLetters feature to suggest their own rules and comments on this important subject.

## Rule 1: Do Not Accept a Review Assignment unless You Can Accomplish the Task in the Requested Timeframe—Learn to Say No

Late reviews are not fair to the authors, nor are they fair to journal staff. Think about this next time you have a paper under review and the reviewers are unresponsive. You do not like delays when it is your paper, neither do the authors of the paper you are reviewing. Moreover, a significant part of the cost of publishing is associated with chasing reviewers for overdue reviews. No one benefits from this process.

## Rule 2: Avoid Conflict of Interest

Reviews come in various forms—anonymous, open, and double-blind, where reviewers are not revealed to the authors and authors are not revealed to reviewers. Whatever the process, act accordingly and with the highest moral principles. The cloak of anonymity is not intended to cover scientific misconduct. Do not take on the review if there is the slightest possibility of conflict of interest. Conflicts arise when, for example, the paper is poor and will likely be rejected, yet there might be good ideas that you could apply in your own research, or, someone is working dangerously close to your own next paper. Most review requests first provide the abstract and then the paper only after you accept the review assignment. In clear cases of conflict, do not request the paper. With conflict, there is often a gray area; if you are in any doubt whatsoever, consult with the Editors who have asked you to review.

## Rule 3: Write Reviews You Would Be Satisfied with as an Author

Terse, ill-informed reviews reflect badly on you. Support your criticisms or praise with concrete reasons that are well laid out and logical. While you may not be known to the authors, the Editor knows who you are, and your reviews are maintained and possibly analyzed by the publisher's manuscript tracking system. Your profile as a reviewer is known by the journal—that profile of review quality as assessed by the Editor and of timeliness of review should be something you are proud of. Many journals, including this one, provide you with the reviews of your fellow reviewers after a paper is accepted or rejected. Read those reviews carefully and learn from them in writing your next review.

## Rule 4: As a Reviewer You Are Part of the Authoring Process

Your comments, when revisions are requested, should lead to a better paper. In extreme cases, a novel finding in a paper on the verge of rejection can be saved by (often) multiple rounds of revision based on detailed reviewers' comments and become highly cited. You are an unacknowledged partner in the success of the paper. It is always beneficial to remember that you are there to help the authors in their work, even if this means rejecting their manuscript.

## Rule 5: Be Sure to Enjoy and to Learn from the Reviewing Process

Peer review is an important community service and you should participate. Unfortunately, the more you review, in all likelihood the more you will be asked to review. Often you will be asked to review boring papers that are of no interest to you. While it is important to serve as a reviewer, only accept papers in which you are keenly interested, because either they are close to your area of research or you feel you can learn something. You might say, should I not know the work very well to be a reviewer? Often a perspective from someone in a slightly different area can be very effective in improving a paper. Do not hesitate to indicate to the Editor the perspective that you can bring to a paper (see Rule 10); s/he can then decide how to weigh your review. Editors would of course like to see you review papers even if you are not particularly interested in them, but the reality is that good reviewers must use their reviewing time wisely.

## Rule 6: Develop a Method of Reviewing That Works for You

This may be different for different people. A sound approach may be to read the manuscript carefully from beginning to end before considering the review. This way you get a complete sense of the scope and novelty of the work. Then read the journal's Guide to Authors, particularly if you have not published in the journal yourself, or if the paper is a particular class of article with which you are not overly familiar, a review for example. With this broad background, you can move to analyzing the paper in detail, providing a summary statement of your findings as well as detailed comments. Use clear reasoning to justify each criticism, and highlight the good points about the work as well as the weaker points. Including citations missed by the author (not your own) is often a short but effective way to help improve a paper. A good review touches on both major issues and minor details in the manuscript.

## Rule 7: Spend Your Precious Time on Papers Worthy of a Good Review

The publish-or-perish syndrome leads to many poor papers that may not be filtered out by the Editors prior to sending it out for review. Do not spend a lot of time on poor papers (this may not be obvious when you take on the paper by reading only the abstract), but be very clear as to why you have spent limited time on the review. If there are positive aspects of a poor paper, try to find some way of encouraging the author while still being clear on the reasons for rejection.

## Rule 8: Maintain the Anonymity of the Review Process if the Journal Requires It

Many of us have received reviews where it is fairly obvious who reviewed the work, sometimes because they suggest you cite their work. It is hard to maintain anonymity in small scientific communities, and you should reread your review to be sure it does not endanger the anonymity if anonymous reviews are the policy of the journal. If anonymity is the rule of the journal, do not share the manuscript with colleagues unless the Editor has given the green light. Anonymity as a journal policy is rather a religious rule—people are strongly for and against. Conform strictly to the policy defined by the journal asking you to review.

## Rule 9: Write Clearly, Succinctly, and in a Neutral Tone, but Be Decisive

A poorly written review is as bad as a poorly written paper (see Rule 3). Try to be sure the Editors and the authors can understand the points you are making. A point-by-point critique is valuable since it is easy to read and to respond to. For each point, indicate how critical it is to your accepting the paper. If English is not your strong point, have someone else read the paper and the review, but without violating other rules, particularly Rule 2. Further, as passionate as you might be about the subject of the paper, do not push your own opinion or hypotheses. Finally, give the Editors a clear answer as to your recommendation for publication. Reviewers frequently do not give a rating even when requested. Provide a rating—fence-sitting prolongs the process unnecessarily.

## Rule 10: Make Use of the “Comments to Editors”

Most journals provide the opportunity to send comments to the Editors, which are not seen by the authors. Use this opportunity to provide your opinion or personal perspective of the paper in a few clear sentences. However, be sure those comments are clearly supported by your review—do not leave the Editor guessing with comments like “this really should not be published” if your review does not strongly support that statement. It is also a place where anonymity can be relaxed and reasons for decisions made clearer. For example, your decision may be based on other papers you have reviewed for the journal, which can be indicated in the Editor-only section. It is also a good place to indicate your own shortcomings, biases, etc., with regard to the content of the paper (see Rule 5). This option is used too infrequently and yet can make a great deal of difference to an Editor trying to deal with a split decision.
